# A Comparative Study of Different EEG Reference Choices for Event-Related Potentials Extracted by Independent Component Analysis

**DOI:** 10.3389/fnins.2019.01068

**Published:** 2019-10-11

**Authors:** Li Dong, Xiaobo Liu, Lingling Zhao, Yongxiu Lai, Diankun Gong, Tiejun Liu, Dezhong Yao

**Affiliations:** ^1^MOE Key Laboratory for Neuroinformation, The Clinical Hospital of Chengdu Brain Science Institute, University of Electronic Science and Technology of China, Chengdu, China; ^2^School of Life Science and Technology, Center for Information in Medicine, University of Electronic Science and Technology of China, Chengdu, China

**Keywords:** EEG, event-related potential (ERP), independent component analysis (ICA), reference choices, REST reference

## Abstract

In the event-related potential (ERP) of scalp electroencephalography (EEG) studies, the vertex reference (Cz), linked mastoids or ears (LM), and average reference (AVG) are popular reference methods, and the reference electrode standardization technique (REST) is increasingly applied. Because scalp EEG recordings are considered as spatially degraded signals, independent component analysis (ICA) is a widely used data-driven method for obtaining ERPs by decomposing EEG data. However, the accurate estimation of the differences in ERP components extracted by ICA with different references remains unclear. In this study, we first provided formal descriptions of the above reference methods (Cz, LM, AVG, and REST) and ICA decomposition in ERP and then investigated the influences of different reference techniques on simulation and real EEG datasets. The results revealed that (1) the reference method did not change the peak amplitudes and latencies of relative ERPs corresponding to some IC time courses; (2) there were non-negligible effects of different reference methods on both temporal ERPs and spatial topographies of some ICs; and (3) compared to Cz, LM, and AR, considering both the performances of temporal ERPs and spatial topographies, the REST reference had overall superiority. These findings provide a recommended choice of REST for ICA analysis at the trial level and contribute to empirical investigations regarding the use of reference methods in ERP domains with ICA analysis.

## Introduction

Since the first report in 1929 (Berger, 1929), scalp electroencephalography (EEG) has been a cost-effective and non-invasive technique that directly quantifies the mean electrical activity of the brain at scalp sites with excellent temporal resolution (~milliseconds) (Cohen, 2017). In addition, event-related potentials (ERPs) are one of the most widely used EEG measures to assess brain activity in response to specific sensory, motor or cognitive events, and several ERP components (e.g., N1, P2, and P3) can provide comprehensive information about low or high level cognitive functions of the brain (Luck, 2014). Scalp ERP has remained one of most informative measures in clinical neurophysiology (Johnstone et al., 2013; Li et al., 2019a; Qin et al., 2019) and human cognitive neuroscience (Enriquez-Geppert et al., 2017; Tian et al., 2018; Li et al., 2019b) research for several decades.

In the scalp EEG domain, because of the volume conduction effect, there is no point on the body or head where the reference potential is ideal zero or constant (Dien, 1998; Yao, 2001, 2017). In the cognitive ERP domain, this physical phenomenon is known as the “no-Switzerland principle” (Luck, 2014): there is no electrically neutral site on the body or head, and Switzerland is politically neutral. Obviously, for evaluating the characteristics (e.g., connectivity, latency, and voltage amplitudes) of EEG accurately at the trial level, it is necessary to use an appropriate reference method to minimize the potential effects of the reference on the results. Currently, a number of different reference methods have been proposed, including the vertex reference (Cz) (Lehmann et al., 1998), linked mastoids or ears (LM) (Gevins and Smith, 2000), average reference (AVG) (Offner, 1950) and reference electrode standardization technique (REST) (Yao, 2001; Dong et al., 2017). Under their certain assumptions, reference methods including Cz, LM, and AVG have been commonly used while ignoring that they are not zero references. As a novel method that approximately converts an average or unipolar reference to a zero reference (Yao, 2001; Dong et al., 2017), REST is increasingly acknowledged by EEG research groups around the world and applied in various EEG studies (Mumtaz et al., 2017a,b; Tian et al., 2017; Li et al., 2019a). Moreover, the above different references have been investigated in many comparative EEG studies, including EEG spectrum (Yao et al., 2005; Chella et al., 2014, 2017), EEG coherence (Marzetti et al., 2007), ERPs (Tian and Yao, 2013; Liu et al., 2015; Hu et al., 2017; Mahajan et al., 2017; Qin et al., 2017; Yang et al., 2017; Li et al., 2018) and brain network analyses (Qin et al., 2010; Chella et al., 2016; Lei and Liao, 2017), and the merit of the REST reference has been confirmed. However, at present, the choice of appropriate EEG references in EEG studies across the world remains an open issue, especially in the ERP domain (Luck, 2014).

Due to volume conduction, scalp EEG/ERP recordings can be considered spatially degraded signals, which reflect spatial and temporal mixtures of the electrophysiological signals of multiple independent sources/components that are generated from large-scale synchronous field potentials (Onton and Makeig, 2006; Winter et al., 2007). Because of the EEG mixing problem, blind source separation via independent component analysis (ICA) (Hyvarinen and Oja, 2000) has been one of the most popular methods for decomposing EEG data. On the one hand, ICA can be used to separate artifact components (such as eye blinks, eye movement, facial muscles, and ballistocardiogram) from raw separately or simultaneously recorded EEG data and then reconstruct clean EEG signals (Srivastava et al., 2005; Hoffmann and Falkenstein, 2008). On the other hand, ICA is also utilized to demix EEG data to extract ERP components at the trial level, thereby increasing the signal-to-noise ratio (SNR) of ERPs (Delorme et al., 2012; Dong et al., 2014; Lee et al., 2016). In addition, ICA was also used to derive filter coefficients in re-referencing of intracranial EEG (Michelmann et al., 2018). Furthermore, for overcoming the limitations of ICA at the single subject level (it is not naturally suited to match independent components from each subject to allow group inferences), group level ICA is further proposed to aggregate component information across a group of subjects for decomposition (Eichele et al., 2011) and is commonly used for event-related separate or simultaneous EEG data (Lei et al., 2012; Huster et al., 2015; Huster and Raud, 2018; Labounek et al., 2018). Notably, in the most current EEG/ERP studies (Eichele et al., 2011; Labounek et al., 2018), using average referenced data to perform ICA at the trial level is suggested, while average referencing results in zero total potential for all ICA topographies. However, the potential reason for this issue and the potential effects of reference methods on ICA decomposition remain unclear. In addition, although different reference methods, including Cz, LM, AVG, and REST, have been investigated in many ERP studies (Tian and Yao, 2013; Liu et al., 2015; Hu et al., 2017; Mahajan et al., 2017; Qin et al., 2017; Yang et al., 2017; Li et al., 2018), most of them use EEG data analysis without ICA decomposition, and the accurate estimation of the differences in ERP components extracted by ICA with different references remains unclear.

Given that there are no direct comparisons of the effects of different reference methods on ERPs extracted by ICA, the aim of this study was to directly evaluate the choice influence of Cz, LM, AVG, and REST on ICA components at the trial level. The current study not only provided formal descriptions of potential relations or differences between re-referencing and ICA decomposition but also investigated the effects of different reference techniques on one simulation and one real EEG dataset. We assumed that compared to Cz, LM, and AR, REST would be closer to the true case and provide a superior choice for ICA analysis at the trial level.

## Materials and Methods

### Theory

Here, we introduce unified matrix representations of ICA decomposition and various reference methods. Considering ideal EEG recordings of ERPs *V*^*Inf*^ (true infinite reference) with *N* channels, *K* sources, *M* trials, and *T* time points.

(1) $$\begin{array}{lll} V^{Inf} & = & \left[\begin{array}{cccccccccc} v_{11}^{1} & \cdot \cdot \cdot & v_{1T}^{1} & v_{21}^{1} & \cdot \cdot \cdot & v_{2T}^{1} & \cdot \cdot \cdot & v_{M1}^{1} & \cdot \cdot \cdot & v_{MT}^{1}\\ v_{11}^{2} & \cdot \cdot \cdot & v_{1T}^{2} & v_{21}^{2} & \cdot \cdot \cdot & v_{2T}^{2} & \cdot \cdot \cdot & v_{M1}^{2} & \cdot \cdot \cdot & v_{MT}^{2}\\ \vdots & \; & \; & \; & \ddots & \; & \; & \; & \; & \vdots \\ v_{11}^{N} & \cdot \cdot \cdot & v_{1T}^{N} & v_{21}^{N} & \cdot \cdot \cdot & v_{2T}^{N} & \cdot \cdot \cdot & v_{M1}^{N} & \cdot \cdot \cdot & v_{MT}^{N} \end{array}\right]\\ \; & = & L_{N\times K}S_{K\times K_{2}}P_{K_{2}\times MT}+\varepsilon \end{array}$$

where *N* is the channel number; *K* is the number of sources/voxels; *K*_2_ is the number of ERPs; *M* is the number of ERP trials; *T* is the number of time points for each trial; $v_{ij}^{k},1\leq k\leq N,1\leq i\leq M,1\leq j\leq T$ is a sample at *k*th channel, *i*th trial and *j*th sample point; *L* is the lead-field matrix; *S* is true spatial sources; *LS* is the true spatial topographies; *P* is true temporal potentials of ERPs; and ε is a noise item.

For a unipolar reference with *k*th-channel (e.g., Cz) as a reference, the referenced ERP signal can be

(2) $$\begin{array}{lll} V^{Cz} & = & V^{Inf}-\left[0,\cdot \cdot \cdot 0,1^{Cz},0,\cdot \cdot \cdot 0_{N}\right]V^{Inf}\\ \; & = & \left(I-{\tilde{T}}^{Cz}\right)V^{Inf}\\ \; & = & T^{Cz}V^{Inf}\\ \; & = & T^{Cz}L_{N\times K}S_{K\times K_{2}}P_{K_{2}\times MT}+T^{Cz}\varepsilon \end{array}$$

where *I* is a unit matrix with 1 in the diagonal, ${\tilde{T}}^{Cz}$ is a Cz transform matrix, and *T*^*Cz*^ is the transform matrix from the ideal ERPs to the actual ERP reference.

Similarly, for LM and average references, we have the formulas for referenced ERP signals

(3) $$\begin{array}{lll} V^{LM} & = & V^{Inf}-\frac{1}{2}\left[\begin{array}{ccccccccc} 0 & \cdot \cdot \cdot & 1_{j_{1}} & \cdot \cdot \cdot & 0 & \cdot \cdot \cdot & 1_{j_{2}} & \cdot \cdot \cdot & 0_{N} \end{array}\right]V^{Inf}\\ \; & = & \left(I-{\tilde{T}}^{LM}\right)V^{Inf}\\ \; & = & T^{LM}V^{Inf}\\ \; & = & T^{LM}L_{N\times K}S_{K\times K_{2}}P_{K_{2}\times MT}+T^{LM}\varepsilon \end{array}$$

(4) $$\begin{array}{lll} V^{AVG} & = & V^{Inf}-\left[\begin{array}{ccc} \frac{1}{N} & \cdot \cdot \cdot & \frac{1}{N}\\ \vdots & \ddots & \vdots \\ \frac{1}{N} & \cdot \cdot \cdot & \frac{1}{N} \end{array}\right]\;V^{Inf}\\ \; & = & \left(I-{\tilde{T}}^{AVG}\right)V^{Inf}\\ \; & = & T^{AVG}V^{Inf}\\ \; & = & T^{AVG}L_{N\times K}S_{K\times K_{2}}P_{K_{2}\times MT}+T^{AVG}\varepsilon \end{array}$$

where *T*^*LM*^ and *T*^*AVG*^ are the transform matrix from the ideal recordings *V*^*Inf*^ to the linked-mastoids/ears and average reference *V*^*LM*^ and *V*^*AVG*^, respectively.

As an example, considering the scalp EEG recordings of ERPs with average reference, *V*^*AVG*^, the potential with the REST reference (i.e., estimated infinite reference), *V*^*REST*^, can be obtained as follows:

(5) $$\begin{array}{lll} V^{REST} & = & L_{N\times K}S_{K\times K_{2}}P_{K_{2}\times MT}+\varepsilon \\ \approx L\left(\tilde{SP}\right) & = & LL_{AVG}^{+}V^{AVG}\\ \; & = & LL_{AVG}^{+}\left(T^{AVG}LSP+T^{AVG}\varepsilon \right)\\ \; & = & LL_{AVG}^{+}T^{AVG}L_{N\times K}S_{K\times K_{2}}P_{K_{2}\times MT}+LL_{AVG}^{+}T^{AVG}\varepsilon \end{array}$$

where $\overset{\sim }{SP}$ is the estimate of reconstructed equivalent sources with temporal potentials, $L_{AVG}^{+}$ is the Moore-Penrose generalized inverses of lead-field matrix *L, V*^*AVG*^ is average referenced ERP signals, and *T*^*AVG*^ is the transform matrix from the ideal recordings to the average reference.

Thus, for ICA decompositions of the true infinite, Cz, LM, AVG, and REST references, we have the formulas

(6) $$\begin{array}{lll} V^{Inf} & = & G_{N\times K_{1}}^{Inf}{\hat{P}}_{K_{1}\times MT}^{Inf}+\varepsilon_{ICA}^{Inf}\\ \; & = & \left(L_{N\times K}S_{K\times K_{2}}\right)P_{K_{2}\times MT}+\varepsilon \end{array}$$

(7) $$\begin{array}{lll} V^{Cz} & = & G_{N\times K_{1}}^{Cz}{\hat{P}}_{K_{1}\times MT}^{Cz}+\varepsilon_{ICA}^{Cz}\\ \; & = & T^{Cz}L_{N\times K}S_{K\times MT}+T^{Cz}\varepsilon \\ \; & = & \left(T^{Cz}L_{N\times K}S_{K\times K_{2}}\right)P_{K_{2}\times MT}+T^{Cz}\varepsilon \end{array}$$

(8) $$\begin{array}{lll} V^{LM} & = & G_{N\times K_{1}}^{LM}{\hat{P}}_{K_{1}\times MT}^{LM}+\varepsilon_{ICA}^{LM}\\ \; & = & T^{LM}L_{N\times K}S_{K\times MT}+T^{LM}\varepsilon \\ \; & = & \left(T^{LM}L_{N\times K}S_{K\times K_{2}}\right)P_{K_{2}\times MT}+T^{LM}\varepsilon \end{array}$$

(9) $$\begin{array}{lll} V^{AVG} & = & G_{N\times K_{1}}^{AVG}{\hat{P}}_{K_{1}\times MT}^{AVG}+\varepsilon_{ICA}^{AVG}\\ \; & = & T^{AVG}L_{N\times K}S_{K\times MT}+T^{AVG}\varepsilon \\ \; & = & \left(T^{AVG}L_{N\times K}S_{K\times K_{2}}\right)P_{K_{2}\times MT}+T^{AVG}\varepsilon \end{array}$$

(10) $$\begin{array}{lll} V^{REST} & = & L_{N\times K}S_{K\times K_{2}}P_{K_{2}\times MT}+\varepsilon \approx G_{N\times K_{1}}^{REST}{\hat{P}}_{K_{1}\times MT}^{REST}+\varepsilon_{ICA}^{REST}\\ \; & = & LL_{AVG}^{+}T^{AVG}L_{N\times K}S_{K\times K_{2}}P_{K_{2}\times MT}+LL_{AVG}^{+}T^{AVG}\varepsilon \\ \; & = & \left(LL_{AVG}^{+}T^{AVG}L_{N\times K}S_{K\times K_{2}}\right)P_{K_{2}\times MT}+LL_{AVG}^{+}T^{AVG}\varepsilon \end{array}$$

where G is the *N*-by-*K*_1_ ICA mixing matrix (corresponding to the spatial topographies), $\hat{P}$ are the *K*_1_-by-*MT* independent component time courses (corresponding to the ERPs), *P* is true ERPs and ε is ICA error or a recording noise item. Notably, ICA mixing matrix G does not have to equal the final transform matrix from true potentials *P* to scalp recordings *V* (e.g., *K*_1_ does not have to equal to *K*_2_ in some cases). In this work, the potential effects of different EEG reference choices for ERPs extracted by ICA are investigated.

### Simulation

**Figure 1:**
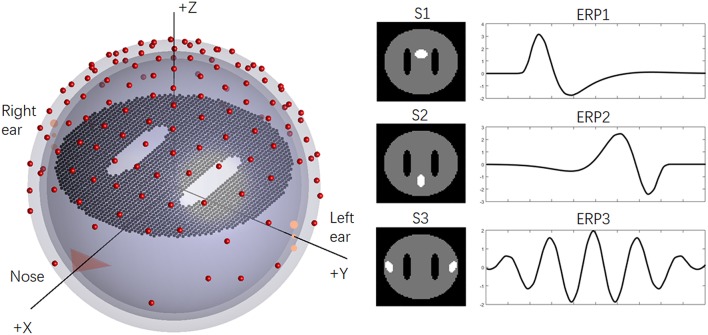
The basic simulation of the head model and sources. On the left, the simplified concentric three-sphere head model; on the right, the source spatial distributions (S1–S3) and corresponding ERPs (ERP1-ERP3).

To illustrate the previous questions, we employed a disc (one slice) with 2,452 dipoles to generate the simulation EEG data, and “white matter” regions were represented by two holes in the disc. For the basic ERP setup, a concentric three-sphere head model with 129 electrodes was used, and the analytic solution sphere radii were set as [0.87 0.89 1]. The orientations of the ERP sources (dipoles) were fixed as +z axis, and the lead-field matrix was calculated analytically (Yao et al., 2004). The temporal sampling rate of the EEG was typically 1 kHz. As an example, a total of 100 target stimuli (trials) were assumed to be collected from EEG data. The epoch of the ERPs was 1,000 ms, which consisted of 200 time points after downsampling to 200 Hz. Three sources were implemented and drawn with white color on the disc: “anterior cingulate area,” “precuneus area,” and “auditory cortex” (abbreviated as S1–S3). More details regarding the setups can be seen in Figure 1.

Different Gaussian noise with independent and identical distributions (IID) was added to EEG data in the abovementioned simulations, and a conservative signal-to-noise ratio was set at 1. Then, the infinite (Inf) EEG data of all trials (129 channels × 20,000 points) were re-referenced to Cz, LM, AVG, and REST references, and Infomax ICA was used to decompose the re-referenced EEG data (the number of ICs was fixed at 3, i.e., *K*_1_ = *K*_2_ = 3). Next, independent component time courses (i.e., ERPs) were averaged across trials and standard z-scored across time points. These settings and processes were consistent with typical experimental EEG data and analysis (Lei et al., 2010; Dong et al., 2014). Performances of ICA time courses were quantified using root mean square error (RMSE) between the true noiseless ERPs (i.e., *P* in Equation 1) and ICA-extracted ERPs (i.e., $\hat{P}$ in Equations 6–10) with a reference (Cz, LM, AVG, REST, or Inf). Performances of ICA spatial topographies were quantified using Pearson's correlation between the true topographies (i.e., *LS* in Equation 1) and ICA-extracted topographies (*G* in Equations 6–10) with a reference (Cz, LM, AVG, REST, or Inf). The whole simulation process was repeated 100 times to obtain mean results, and the effects of different EEG references for ERPs and topographies extracted by ICA were analyzed using one-way analysis of variance (ANOVA, *p* < 0.001) and a *post-hoc* test (Tukey's honest significant difference criterion, *p* < 0.001). In addition, a range of SNRs from 0.1 to 20 (0.1, 0.2, 0.4, 0.6, 0.8, 1, 1.5, 2, 2.5, 5, 10, and 20) was investigated.

### Real Data

#### Participants and Experiment

In this work, a total of 12 healthy right-handed participants with no history of psychiatric or neurological disorders participated in the experiment after providing written informed consent (10 men and 2 women, mean age: 24.0 years, age range: 20–27 years). An experimental go/no-go task of auditory stimuli was utilized, and sounds were generated by SONAR 6 Producer Edition. The stimulus was presented in 8 blocks (120 trials each) in a random order, and all stimuli were matched for duration and pitch by using Adobe Audition 3.0. All subjects were exposed to a stimulus tone (375 ms in duration, C6/1,046 Hz, 80 ms rise and fall time, American notation) and a loud or soft (58 or 55 dB) timbre piano or violin and were instructed to make a left or right hand (piano or violin) response to a loud violin (go trials, probability 67%) and no response to a soft violin (no-go trials, probability 33%). The higher probability for go trials was chosen to encourage participants to initiate response preparation (Low and Miller, 1999). For each trial, a cross was first presented to instruct the subject to concentrate their attention on the middle of the screen, and a flashing cross was subsequently presented for 500 ms to remind the subject of the upcoming tone (lasting 375 ms). The subject was instructed to respond quickly and accurately in the go trials by pressing a sequence of three keys (the comma, period and slash keys for the right hand, and the C, X, and Z keys for the left hand) with the index, middle and ring fingers. For no-go trials, subjects were instructed to avoid any response. More details of the experimental task can be found in the relevant article (Gong et al., 2012). The study was also approved by the local Ethics Committee of University of Electronic Science and Technology of China (UESTC) in accordance with the standards of the Declaration of Helsinki.

#### EEG Recording

**Figure 2:**
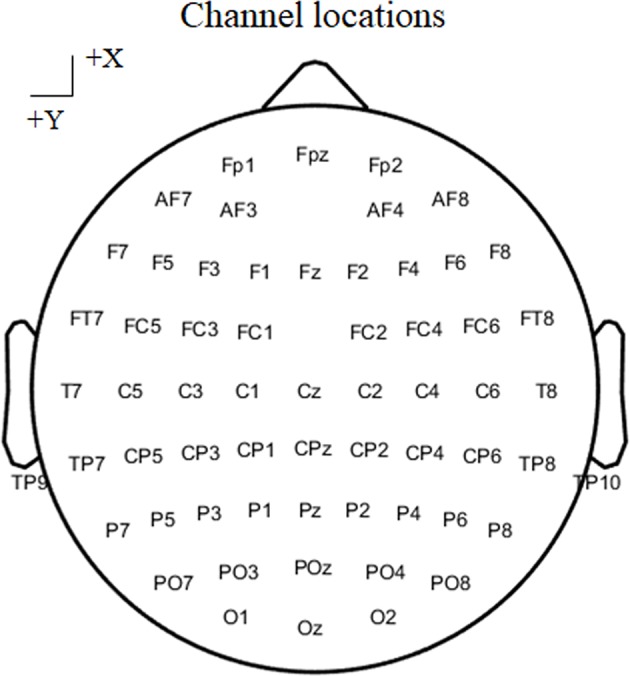
The position of channels according to the 10–20 cap system in real EEG data.

EEG data were recorded using a 64-channel EEG system (Brain Products GmbH, Gilching, Germany). The sampling rate was set at 500 Hz, the FCz served as the recording reference, and the AFz served as the ground electrode. Sixty-one electrodes according to the 10–20 cap system were used to record EEG data (Figure 2), and two channels were utilized to record vertical and horizontal EOG data. The impedance for all channels was maintained below 5 KΩ, and EEG data were bandpass filtered between 0.01 and 100 Hz. During stimulus presentation and response, subjects were asked to remain relaxed and avoid eye blinking.

#### EEG Data Processing

In this work, only the go trials with the loud violin are reported. A pipeline tool (WB_EEG_CalcERP) in WeBrain (a cloud computing platform, http://webrain.uestc.edu.cn/) was used to obtain go trials with the loud violin from raw EEG data, and EEGIFT (v2.5, http://trendscenter.org/software/eegift/#) was used to extract ERPs by group ICA. In brief, EEG channels were visually inspected first, and no bad channels were detected for all subjects. Then, EEG data were preprocessed using the WeBrain pipeline, which includes 1–30 Hz bandpass filtering, data segmentation (−200 to 800 ms), baseline correction (−200 to 0 ms), and the exclusion of artifact-containing trials (exceeding amplitude of ±100 μV, exceeding voltage step/sampling point of 50 μV or exceeding absolute difference of 150 μV in the epoch) and wrong-reaction trials. Then, the preprocessed EEG trials were re-referenced to Cz, LM, AVG, and REST references using REST (Yao, 2001; Dong et al., 2017) (EEGLAB Plugin version v1.0, http://www.neuro.uestc.edu.cn/rest/) and NIT (Dong et al., 2018) (v1.3, http://www.neuro.uestc.edu.cn/NIT.html) tools. Next, ERPs according to each reference were obtained by group Infomax ICA (repeated 20 times using ICASSO). The number of ICs was estimated as 4 using the minimum description length criteria, and the ICA time course was standard *z*-scored across time points.

Considering that true noiseless ERPs in real scalp EEG data were inaccessible and that REST approximately realizes the physical zero reference at infinity (Yao, 2001, 2017; Yao et al., 2019), the performances of ICA time courses were quantified using RMSE between the ERPs with a reference (Cz/LM/AVG) and with the REST reference. Performances of ICA spatial topographies were quantified using Pearson's correlation between the topographies with a reference (Cz/LM/AVG) and with the REST reference. The effects of different EEG references for ERPs and topographies were also investigated using one-way repeated ANOVA (*p* < 0.05) and *post hoc* paired *t*-test (*p* < 0.05).

## Results

### Simulation Results

**Figure 3:**
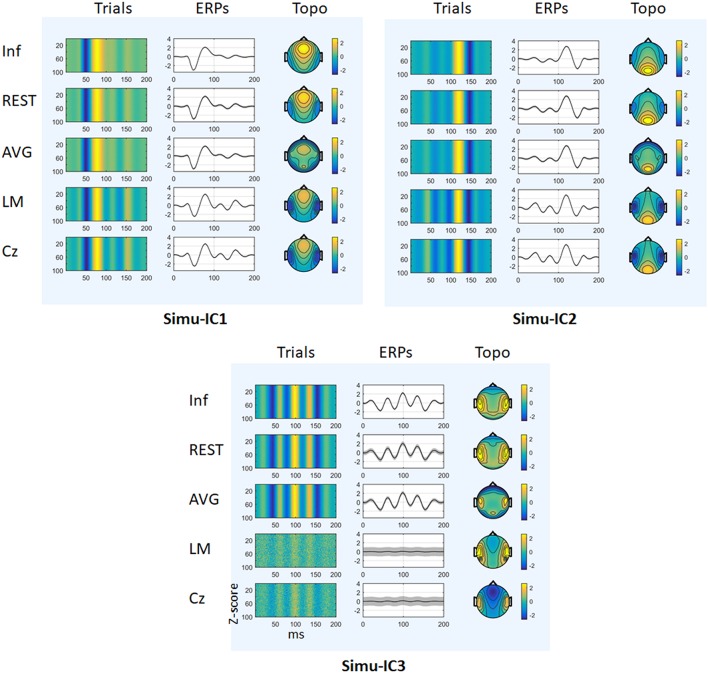
Results of simulation (SNR = 1). The mean trials, ERPs and topographic maps of each ICA component (Simulation IC1–IC3) for the Inf, REST, AVG, LM, and Cz references are shown separately. The gray region in ERPs represents standard deviations across 100 repeats.

**Figure 4:**
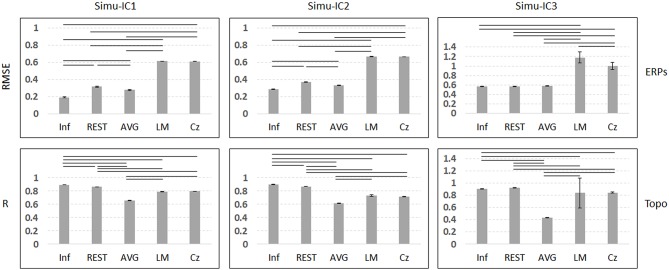
Results of one-way ANOVA (*p* < 0.001) and a *post-hoc* test (black line means *p* < 0.001) for simulation of SNR = 1. Vertical axis of the first row is the mean RMSE (with standard deviation) between true noiseless ERPs and ICA-extracted ERPs with a reference, while the vertical axis of the second row is the mean spatial correlation (with standard deviation) between true topographies and ICA-extracted topographies with a reference. Columns from left to right correspond to the ICA components (Simulation IC1–IC3).

Figure 3 depicts the mean trials, ERPs and topographic maps of ICA components for Inf, REST, AVG, LM, and Cz references separately, while the SNR = 1. ERP1 and ERP2 could be detected by ICA with different choices of references, and the spatial distribution patterns of the spatial components were similar for different references to some degree. ERP3 could be detected by ICA with Inf, REST, and AVG references and could not be well-detected with LM and Cz references. Furthermore, one-way ANOVA and a *post-hoc* test were performed to quantify the performances of ICA time courses (RMSEs between true noiseless ERPs and ICA-extracted ERPs with a reference) and topographies (spatial correlations between true topographies and ICA-extracted topographies with a reference). There were significant differences [Simu-IC1: *F*_(4, 495)_ = 1.77 × 10^5^, *p* < 0.001; Simu-IC2: *F*_(4, 495)_ = 4.97 × 10^5^, *p* < 0.001; Simu-IC3: *F*_(4, 495)_ = 2.19 × 10^3^, *p* < 0.001] of RMSEs with different references (Figure 4, first row). For RMSEs of Simu-IC1 and Simu-IC2 (corresponding to ERP1 and ERP2), Tukey's tests revealed significant differences (*p* < 0.001) for almost all pairwise comparisons among these references (except for the comparison between LM and Cz). For Simu-IC3 (corresponding to ERP3), the RMSEs of the LM reference were significantly higher than those of the Inf, REST, AVG, and Cz references, and the RMSEs of the Cz reference were higher than those of the Inf, REST, and AVG references. Significant differences between ICA-extracted topographies [Simu-IC1: *F*_(4, 495)_ = 8.93 × 10^4^, *p* < 0.001; Simu-IC2: *F*_(4, 495)_ = 3.14 × 10^4^, *p* < 0.001; Simu-IC3: *F*_(4, 495)_ = 337.38, *p* < 0.001] with different references were also detected (Figure 4, second row). For the spatial distribution patterns of Simu-IC1 and Simu-IC2 (corresponding to S1 and S2), Tukey's tests revealed significant differences (*p* < 0.001) for almost all pairwise comparisons among these references (except for the comparison between LM and Cz). For spatial distribution patterns of Simu-IC3 (corresponding to S3, *p* < 0.001), the spatial correlations of Inf and REST references were significantly higher than those of AVG, LM, and Cz references, and the correlations of the AVG reference were also lower than those of LM and Cz references.

**Figure 5:**
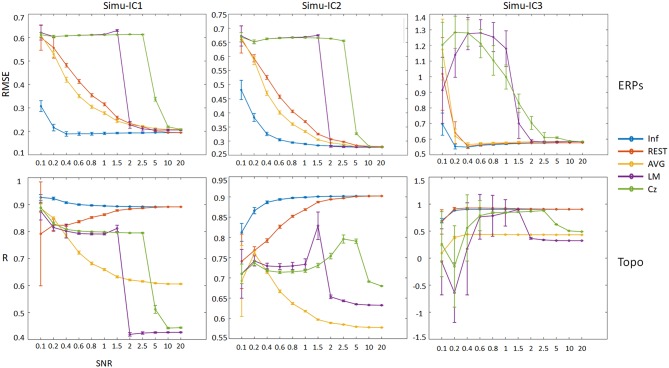
Performances of ICA time courses and topographies with different references (Inf, REST, AVG, LM, and Cz) using a range of SNRs from 0.1 to 20. The vertical axis of the first row is the mean RMSE (with standard deviation) between true noiseless ERPs and ICA-extracted ERPs with a reference, and the vertical axis of the second row is the mean spatial correlation (with standard deviation) between true topographies and ICA-extracted topographies with a reference. Columns from left to right correspond to the ICA components (Simu-IC1, Simu-IC2, and Simu-IC3), and the horizontal axis is the SNR.

In addition, to assess the effects of noise on the performance of the ICA time course and spatial topographies, we considered different SNRs for each reference; the results are shown in Figure 5 (details of ERPs and topographic maps of each ICA component can be seen in Figures S1–S11). For ERPs of Simu-ICs corresponding to ERP1-3, the RMSEs of Inf, REST, and AVG generally decreased as the SNR increased, and the RMSEs of LM and Cz suddenly decreased (cliff-like) with increases in SNR. For the spatial topographies of Simu-IC1, the spatial correlations of REST converged to Inf with increases in SNR, and the correlations of AVG, LM, and Cz decreased. For the spatial topographies of Simu-IC2, the spatial correlations of REST and Inf increased with increases in SNR, and the correlations of LM and Cz increased first and then decreased. For the spatial topographies of Simu-IC3, the correlations of Inf, REST and AVG were constant to some degree, and the correlations of LM and Cz also increased first and then decreased. Considering the amplitudes may be independent across trials, a supplementary simulation of ERP trials with independent amplitudes (uniform distribution from 0.5 to 1.5) were also conducted, and similar results were obtained (Figures S14, S15).

### Real Data Results

**Figure 6:**
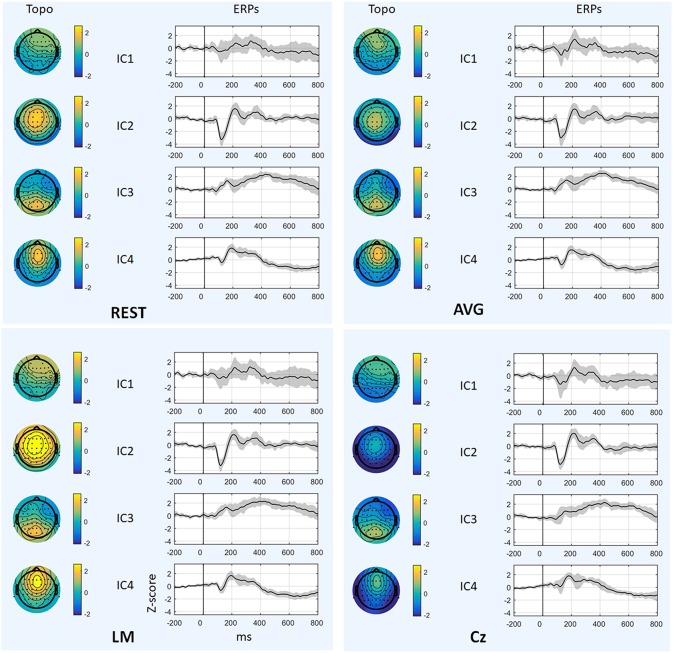
Results of real data. The mean ERPs and topographic maps of each ICA component (IC1–IC4) for REST, AVG, LM, and Cz references are shown separately. The gray region in ERPs represents standard deviation across subjects.

**Figure 7:**
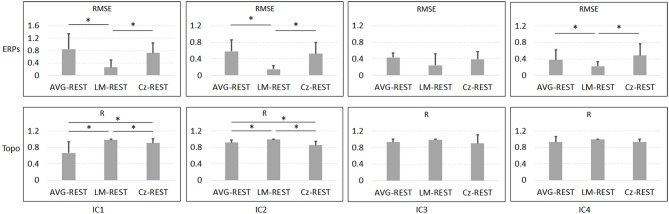
Results of one-way repeated ANOVA (*p* < 0.05) and *post hoc* paired *t*-test (*p < 0.05) for real data. The vertical axis of the first row is the mean RMSE (with standard deviation) between the ICA-extracted ERPs with the AVG/LM/Cz reference and with the REST reference, while the vertical axis of the second row is the mean spatial correlation (with standard deviation) between the ICA-extracted topographies with the AVG/LM/Cz reference and with the REST reference. Columns from left to right correspond to the ICA components (IC1-IC4).

Figure 6 and Table 1 depict the mean ERPs and topographic maps of ICA components of real EEG data for REST, AVG, LM, and Cz references. N1, P2 (indices of sensory processing), and P3 components were detected for all references. Using one-way repeated ANOVA, there were no significant differences among these references for peak and latency values of ICA components (*p* > 0.05). However, there were significant differences [IC2: *F*_(3, 10)_ = 117.63, *p* = 1.11 × 10^−16^; and IC4: *F*_(3, 10)_ = 40.07, *p* = 1.29 × 10^−10^] for pairwise comparisons of the mean values of topographies (mean values of all channels) among these references. For all topographies of ICs, the mean values of the LM reference were the highest, the mean values of REST were second, the mean values of AVG were approximately 0, and the mean values of Cz were negative. Furthermore, one-way repeated ANOVA and *post-hoc* paired *t*-test were performed to quantify the performances of ICA time courses (RMSEs between ICA-extracted ERPs with AVG/LM/Cz and REST references) and topographies (spatial correlations between ICA-extracted topographies with AVG/LM/Cz and REST references). There were significant differences [IC1: *F*_(2, 10)_ = 9.43, *p* = 0.0013; IC2: *F*_(2, 10)_ = 16.36, *p* = 6.17 × 10^−5^; IC4: *F*_(2, 10)_ = 3.90, *p* = 0.037] of RMSEs with different references (Figure 7, first row). For the ERPs of IC1, IC2 (N1, P3a), and IC4 (P2), the RMSEs of the LM reference were significantly lower than those of the AVG and Cz references. Significant differences [IC1: *F*_(2, 10)_ = 12.45, *p* = 3.08 × 10^−4^; IC2: *F*_(2, 10)_ = 17.5, *p* = 4.04 × 10^−5^] of spatial correlations with different references were also detected (Figure 7, second row). For the spatial distribution patterns of IC1 (*p* < 0.05), the spatial correlations of AVG and Cz references were significantly lower than those of the LM reference, and the correlations of the AVG reference were lower than those of the Cz reference. For the spatial distribution patterns of IC2 (*p* < 0.05), the spatial correlations of AVG and Cz references were also significantly lower than those of the LM reference, and the correlations of the AVG reference were higher than those of the Cz reference.

**Table 1:** Details of ERPs and topographic maps extracted by ICA components of real EEG data for REST, AVG, LM, and Cz references (mean ± standard deviation).

			**IC1**	**IC2 (N1)**	**IC2 (P3a)**	**IC3 (P3b)**	**IC4 (P2)**
ERP	Peak (*z*-score)	REST	1.20 ± 1.96	−3.08 ± 2.19	1.46 ± 0.74	2.85 ± 0.36	2.05 ± 0.95
		AVG	1.47 ± 1.92	−2.99 ± 2.18	1.63 ± 0.74	2.96 ± 0.41	1.02 ± 1.78
		LM	1.35 ± 1.90	−2.94 ± 2.14	1.52 ± 0.74	3.05 ± 0.48	1.52 ± 1.59
		Cz	0.79 ± 2.14	−2.61 ± 2.05	1.47 ± 0.59	2.76 ± 0.49	1.57 ± 1.82
	Latency (ms)	REST	291.6 ± 66.3	130.9 ± 24.8	379.8 ± 60.9	404.2 ± 55.0	201.6 ± 39.3
		AVG	265.3 ± 57.9	134.7 ± 25.9	365.8 ± 34.4	392.4 ± 47.2	184.2 ± 44.1
		LM	271.6 ± 58.6	131.8 ± 24.2	387.6 ± 70.7	397.8 ± 55.6	196.0 ± 42.6
		Cz	292.5 ± 72.1	132.7 ± 23.8	371.3 ± 61.5	390.0 ± 58.8	189.8 ± 46.2
Topo	Mean value of all channels	REST	0.20 ± 0.45	0.77 ± 0.32^*^	–	0.27 ± 0.45	0.21 ± 0.30^*^
		AVG	−7.1 × 10^−8^ ± 4.6 × 10^−8^	−9.2 × 10^−8^ ± 3.2 × 10^−8^^*^	–	−4.2 × 10^−9^ ± 4.6 × 10^−8^	−1.4 × 10^−7^ ± 3.2 × 10^−8^^*^
		LM	0.63 ± 1.23	1.99 ± 0.67^*^	–	1.49 ± 1.15	0.93 ± 0.64^*^
		Cz	−0.31 ± 0.54	−1.60 ± 0.58^*^	–	−0.30 ± 0.68	−1.17 ± 0.55^*^

* *There are significant differences for pairwise comparisons among references using one-way repeated ANOVA and post-hoc paired t-test (p < 0.05)*.

## Discussion

In the present study, four commonly used references, Cz, LM, AVG, and REST, were comparatively investigated via the standard ICA analysis of EEG data to reveal the potential effects of different references on ERPs extracted by ICA analysis. The simulation results revealed that the reference approach influenced the IC time courses and corresponding topographic maps, while the SNR = 1. As the SNR increased, the IC time courses of Cz, LM, AVG, and REST converged to Inf; however, only the spatial topographies of REST converged to Inf. Furthermore, we compared the effects of reference methods on the ICs in real EEG data. The results demonstrated that the reference method influenced the most IC time courses but did not change the peak amplitudes and latencies of ERPs corresponding to some IC time courses. In addition, the mean values and distributions of some topographic maps were influenced by reference methods.

### Reference Effects on ICA Performance in Simulations

In this work, conventional simulations were utilized to verify the reference effects on ICA analysis. ICA could well reveal simulation ERP1 and ERP2 with all references and simulation ERP3 with Inf, REST and AVG references. Moreover, for the ERPs of Simu-IC1 and Simu-IC2, Inf obviously introduced the smallest error, and AVG and REST introduced intermediate errors, while LM and Cz references had the largest errors. For the ERPs of Simu-IC3, Inf, REST, and AVG introduced similar errors, and their errors were smaller than those of LM and Cz. These results demonstrated that ICA decomposition could obtain similar event-related potentials from scalp EEG with different references to some degree, and REST and AVG exhibited a performance similar to that of Inf (Figures 2, 3, first row). According to Equations (2)–(5), various reference methods, including Cz, LM, AVG, and REST, could theoretically be presented as unified mathematics (Yao et al., 2019). For Cz, LM, and AVG, the transform matrix (*T*^*Cz*^, *T*^*LM*^, or *T*^*AVG*^) from the ideal ERPs (i.e., Inf) to the corresponding reference (Cz, LM, or AVG) is just a mathematical operation, with its specific assumption, a vertex point of the scalp (e.g., Cz) (Lehmann et al., 1998), the average of the linked mastoids or ears (e.g., LM) (Gevins and Smith, 2000), and the average of all EEG channels (e.g., AVG) (Offner, 1950), which are all non-physical principle-based hypotheses (Yao, 2017). For REST, which is based on the equivalent sources model, head model and electrode montage, the transform matrix *LL*^+^ from the actual ERPs to the REST reference is physically based and reasonable (Yao, 2001, 2017; Yao et al., 2019), and the final transform matrix *LL*^+^*T*^*AVG*^ from Inf ERPs to REST ERPs also has physical meanings. Therefore, all these reference methods can be expressed as unified transform matrix T^**^ (i.e., T^**^P = T^*^LSP) from the ideal temporal potentials of ERPs, *P*, to the corresponding reference recordings, *V*, while *LS* represents the true spatial topographies. As a popular blind source separation method, ICA assumes that signals are Gaussian mixtures containing statistically independent non-Gaussian source time courses (Hyvarinen and Oja, 2000), and the ICA model in ERP decomposition of a single subject can be generally expressed as $V=G\hat{P}$, where *V* represents EEG/ERP signal mixtures corresponding to the reference recordings, *G* is the linearly mixing matrix corresponding to topographies and $\hat{P}$ represents temporal independent components corresponding to ERPs. Noting that ICA decomposition does not have unique solutions, IC time courses are relative changes in ERPs and always are transformed to *z*-scores (Eichele et al., 2011). Therefore, ideally, for some independent event-related potentials in sources, ICA time courses can be relative changes in ERPs, $\hat{P}$, and may proportionally converge (or approximate) to true ERPs (i.e., *P*) in some situations (e.g., for some ERPs with a reference). However, due to the complexity of EEG (e.g., temporal variability across trials, non-stationary, non-linear noise) (Huster et al., 2015; Cohen, 2017) and limitations of ICA (e.g., not suitable for potential non-linear situations, algorithm adaptability, the number of ICs preset may not be appropriate) (Hyvarinen and Oja, 2000; Eichele et al., 2011; Huster et al., 2015), the reference method may have potential influences on the performance of ICA for temporal time courses. Noting that even the number of independent components was set as the true number of ERPs, there were still non-negligible effects of the reference method on ICA time courses.

For spatial topographies of simulation ICs, AVG had the lowest spatial correlations, LM and Cz had intermediate correlations, and Inf had the largest spatial correlations, while REST exhibited a similar performance. These results provide evidence that spatial topographies (i.e., mixing matrix *G* in the ICA model) could be distinctly influenced by different reference methods, and REST performed similarly to Inf (Figures 2, 3, second row). In the ICA model, the spatial topographies are weights that specify the relative contribution of each channel to IC time courses (Hyvarinen and Oja, 2000), and the standard deviation of topographies are usually fixed at 1 (Eichele et al., 2011). The spatial components (e.g., topographies), *G*, may reflect the relative contribution of each channel to ERP waves and relate to the transform matrix T^**^ = T^*^LS corresponding to reference methods in some cases (e.g., Figures S12, S13 show that spatial distributions detected by ICA with REST reference, *G*, were appropriate to transform matrix T^**^ of REST). Because the REST reference is physically based and reasonable (Yao, 2001, 2017; Yao et al., 2019), topographies could be well-detected by ICA with the REST reference. However, because Cz, LM, and AVG are non-zero reference approaches (Yao, 2017) and their transformations of Cz, LM, and AVG (*T*^*Cz*^, *T*^*LM*^, or *T*^*AVG*^) are all seriously singular (Yao et al., 2019), they may influence the mixing matrix of ICA so that the spatial components of ICA have the risk of diverging from the true spatial topographies. Therefore, the effects of reference methods on the topographies of ICs may result in misleading explanations of ERP source generations.

In addition, as the SNR increased, all IC time courses of Cz, LM, AVG, and REST references converged to Inf, and only the spatial topographies of REST converged to Inf (Figure 5 and Figures S1–S11). Meanwhile, considering the amplitudes may be independent across trials, a supplementary simulation of ERP trials with independent amplitudes were conducted, and similar results were obtained (Figures S14, S15). These results provided further evidence that overall, the REST reference had superior performance, which was also consistent with the conclusions of previous simulation studies (Yao, 2001, 2017; Yao et al., 2005; Liu et al., 2015; Chella et al., 2017). Furthermore, for group-level ICA by concatenating subjects (Eichele et al., 2011; Huster and Raud, 2018), REST has a similar unified expression as $\left[\begin{array}{c} V_{1}\\ \vdots \\ V_{Ns} \end{array}\right]=G\left[\begin{array}{c} {\hat{P}}_{1}\\ \vdots \\ {\hat{P}}_{Ns} \end{array}\right]$, while the reference methods can also be expressed as $\left[\begin{array}{c} V_{1}\\ \vdots \\ V_{Ns} \end{array}\right]=T^{**}\left[\begin{array}{c} P_{1}\\ \vdots \\ P_{Ns} \end{array}\right]$, where Ns is the number of subjects. Therefore, similar effects of the reference method on ICA performance may be obtained in this case.

### Reference Effects on ICA in Real Data

To further investigate the potential effects of the reference method on ICA performance, we used real EEG data from an auditory stimulus go/no-go task in this work. A visual inspection of the EEG data showed typical N1, P2, and P3 components corresponding to IC2, IC4, and IC3 in Figure 6 using the Cz, LM, AVG, and REST reference methods, which is also consistent with previous studies (Debener et al., 2005; Eichele et al., 2008; Gong et al., 2012, 2013). Using one-way repeated ANOVA, there were no significant differences among these references for peak and latency values of z-scored ICA components (*p* > 0.05). Considering that true noiseless ERPs in real scalp EEG data were inaccessible and REST approximately realizes the physical zero reference at infinity (Yao, 2001, 2017; Yao et al., 2019), the ERPs of ICA time courses with Cz/LM/AVG and the REST reference were further compared. Additionally, the RMSEs of the LM (LM vs. REST) reference were lower than those of the AVG and Cz references (Figure 7, first row). These results demonstrated that the reference method influenced some IC time courses to some degree but did not change the peak amplitudes and latencies of relative changes of ERPs corresponding to IC time courses.

Because ICA is a data-driven method of matrix decomposition and the mixing matrix specifies the relative contribution of each spatial dimension to IC time courses (Hyvarinen and Oja, 2000), the spatial distributions of topographies may reflect the contribution of each channel to the ERPs of ICs. Because the LM reference is non-zero and the amplitudes of the linked mastoids or ears are contaminated by brain activity (Yao, 2001; Yao et al., 2019), the amplitudes at the active electrodes with LM reference may be increased or decreased by the recorded potentials at the mastoids or ears. Thus, the topographies of some ICs may be influenced by the LM reference, and the Cz reference may lead to similar impacts. Since the potentials of AVG are the average signal of all EEG channels (Offner, 1950), the AVG reference could make all the ICA topographies have zero total potential. Moreover, REST is an approximate zero reference at infinity, which has been proposed as a standard reference method (Yao, 2001, 2017; Yao et al., 2019). In the current study, we compared the mean values of topographies among these references. We found that for IC2 and IC4, the mean values of the LM reference were highest, the mean values of REST were second, the mean values of AVG were ~0, and the mean values of Cz were negative. In addition, even though the topographical distribution revealed similar patterns with all reference methods for most ICs, the topographies of the LM reference were closer to the REST reference for IC1 and IC2 than to the AVG and Cz references (Figure 7, second row). Overall, these results demonstrated that both time courses and spatial distributions of some ICs could be influenced by different reference methods. These findings are consistent with the results of previous ERP comparative studies highlighting the superiority of the REST reference approach (Tian and Yao, 2013; Hu et al., 2017; Mahajan et al., 2017; Qin et al., 2017; Yang et al., 2017; Li et al., 2018; Tian et al., 2018).

## Conclusions

To the best of our knowledge, the present manuscript is the first to investigate the effects of different reference methods on ERPs extracted by ICA analysis over simulation and real EEG datasets. The results of the current study indicated that different EEG reference choices altered both the temporal ERPs and spatial topographies of some ICs. Additionally, compared to Cz, LM, and AR references, the REST reference had overall superiority considering both the performances of temporal ERPs and spatial topographies and could provide a recommended choice for ICA analysis at the trial level. These findings contribute to empirical investigations regarding the use of reference methods in ERP domains with ICA analysis.

## Data Availability Statement

The datasets generated for this study are available on request to the corresponding author.

## Ethics Statement

The studies involving human participants were reviewed and approved by the local Ethics Committee of University of Electronic Science and Technology of China. The patients/participants provided their written informed consent to participate in this study.

## Author Contributions

LD, YL, and DY conceived and designed the work. LD, XL, and LZ wrote the code, analyzed the data, and realized the simulation. LD and DG acquired the EEG data. LD, TL, YL, DG, and DY wrote and revised the manuscript. All authors revised the work for important intellectual content and read and approved the manuscript.

### Conflict of Interest

The authors declare that the research was conducted in the absence of any commercial or financial relationships that could be construed as a potential conflict of interest.
