# Supplementary material for: A Comparative Study of Different EEG Reference Choices for Event-Related Potentials Extracted by Independent Component Analysis
Source: Front Neurosci. 2019 Oct 11;13:1068. doi: 10.3389/fnins.2019.01068 (PMC6798171; doi:10.3389/fnins.2019.01068)
Supplement: Supplementary file 1 [file Table_1.DOCX]

**Supplementary Materials**

Results of simulation with a range of SNR from 0.1 to 20 (SNR=0.1, 0.2, 0.4, 0.6, 0.8, 1, 1.5, 2, 2.5, 5, 10 or 20).


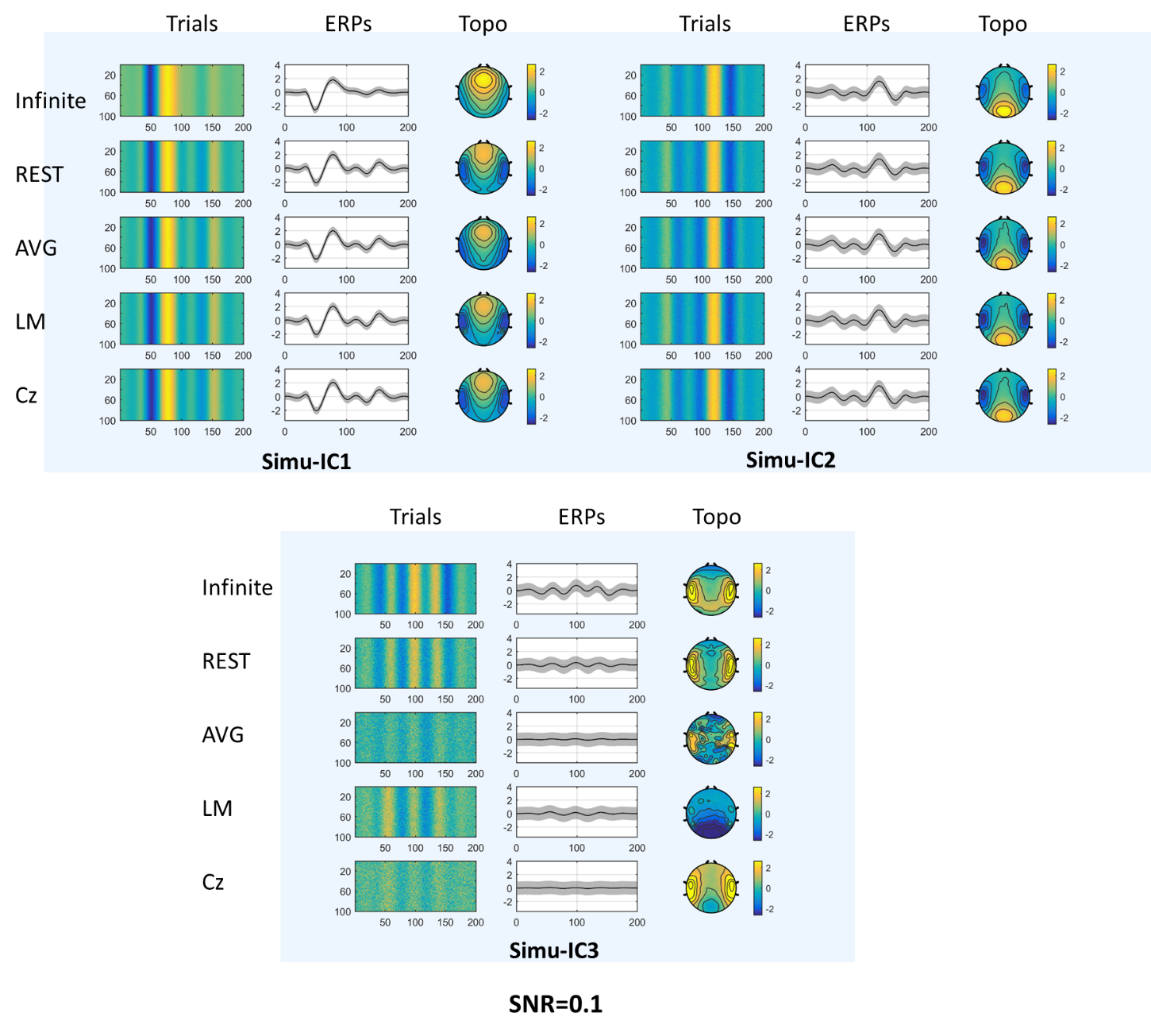


Figure S1: Results of simulation with SNR=0.1


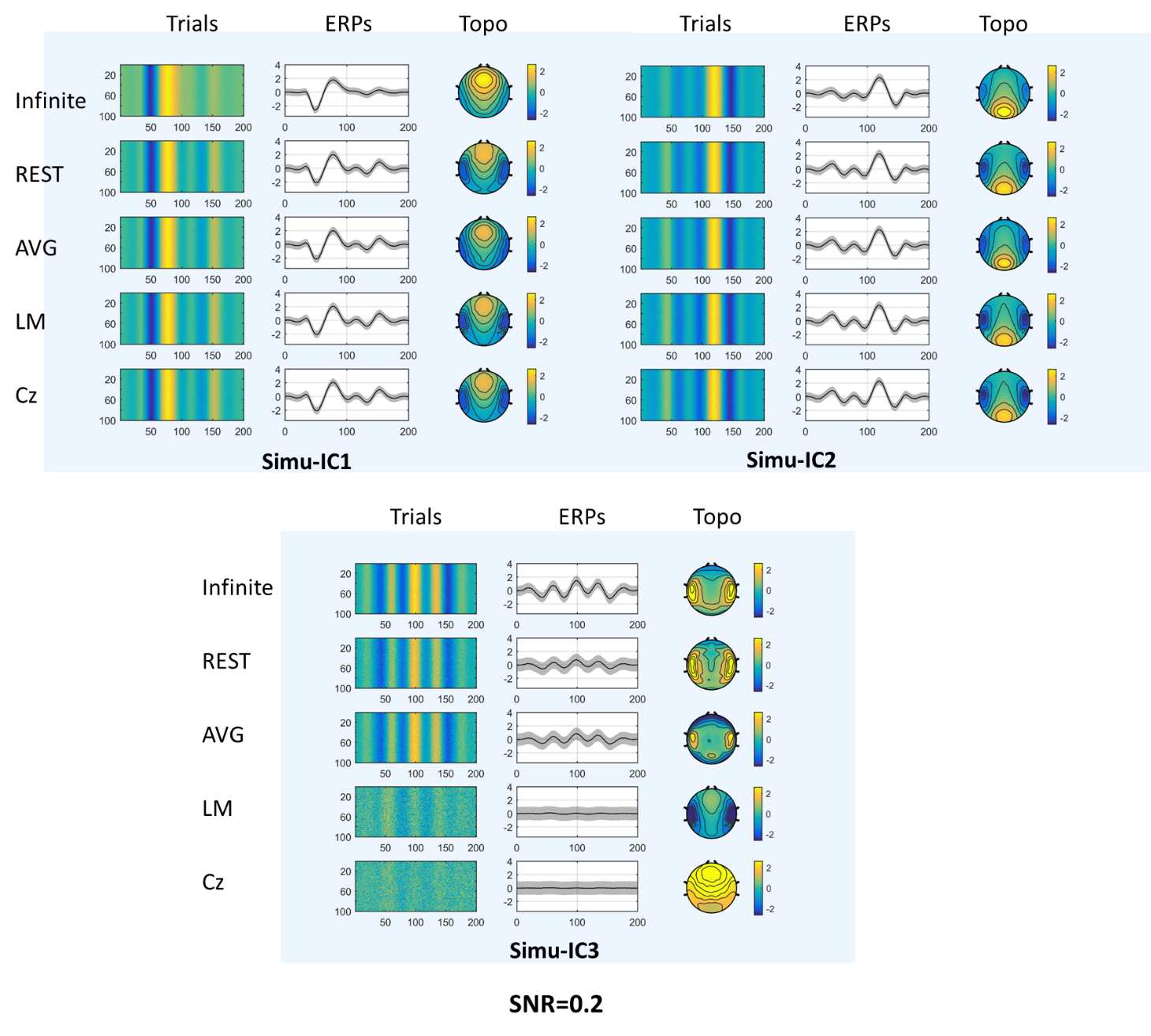


Figure S2: Results of simulation with SNR=0.2


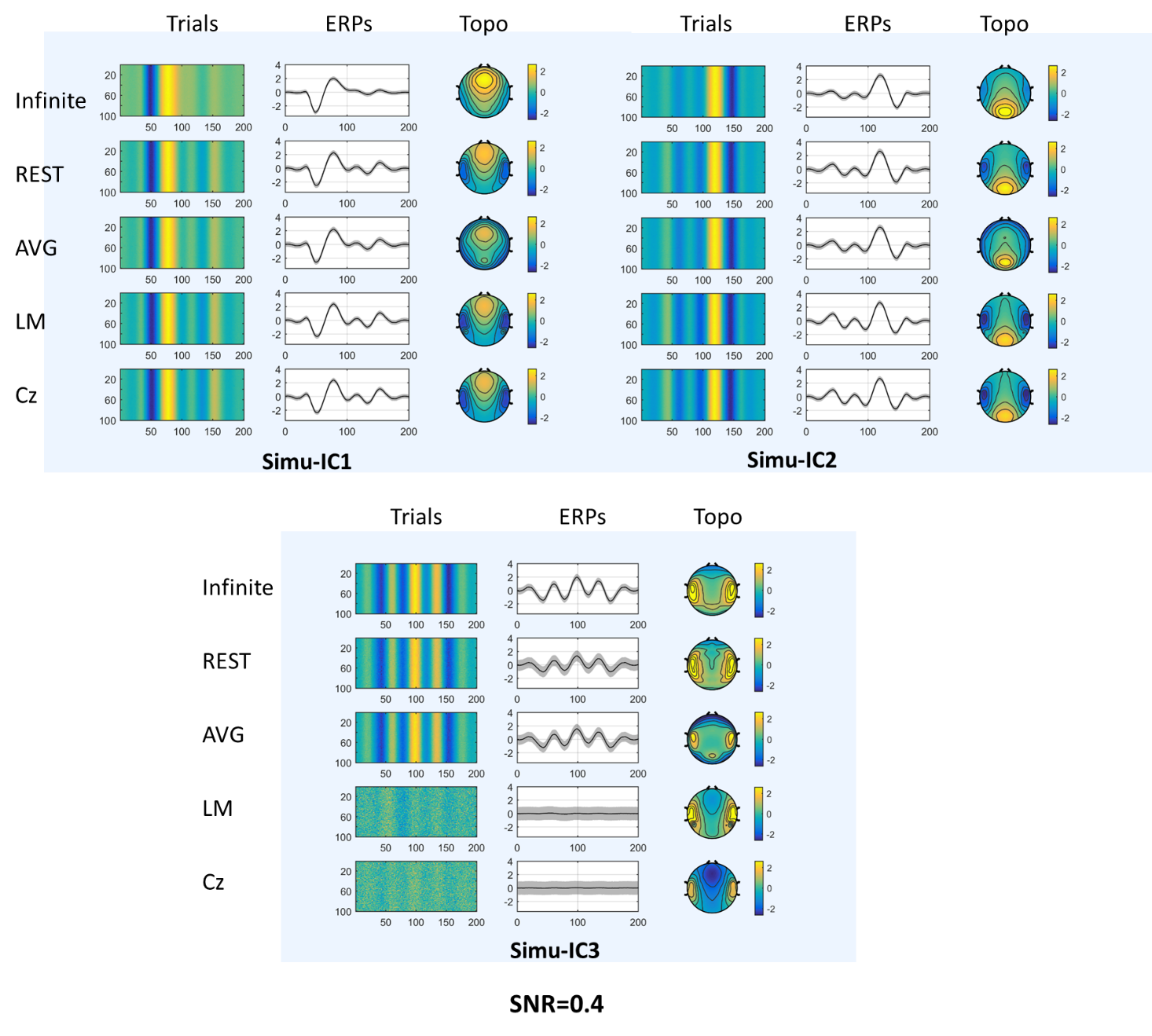


Figure S3: Results of simulation with SNR=0.4


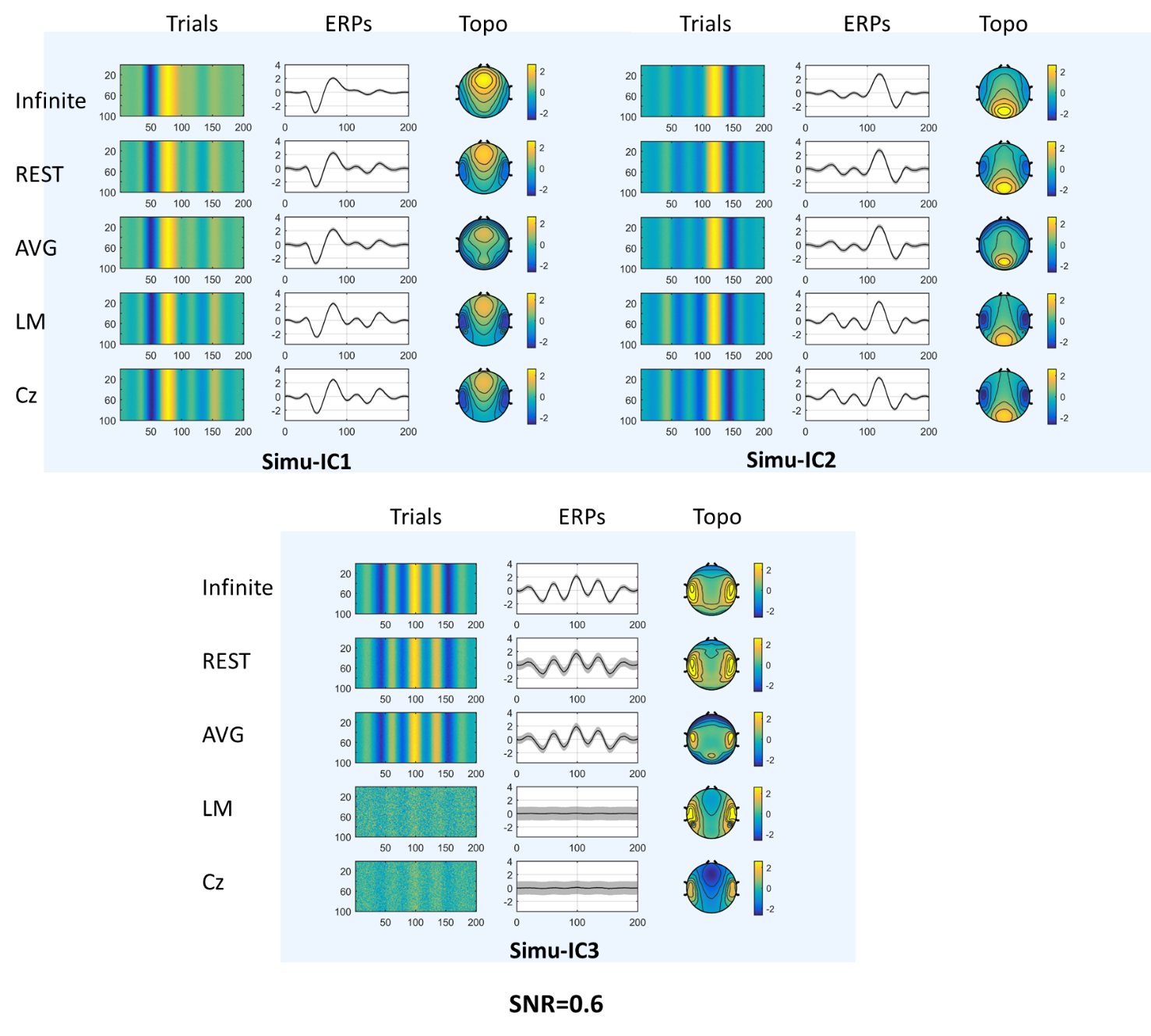


Figure S4: Results of simulation with SNR=0.6


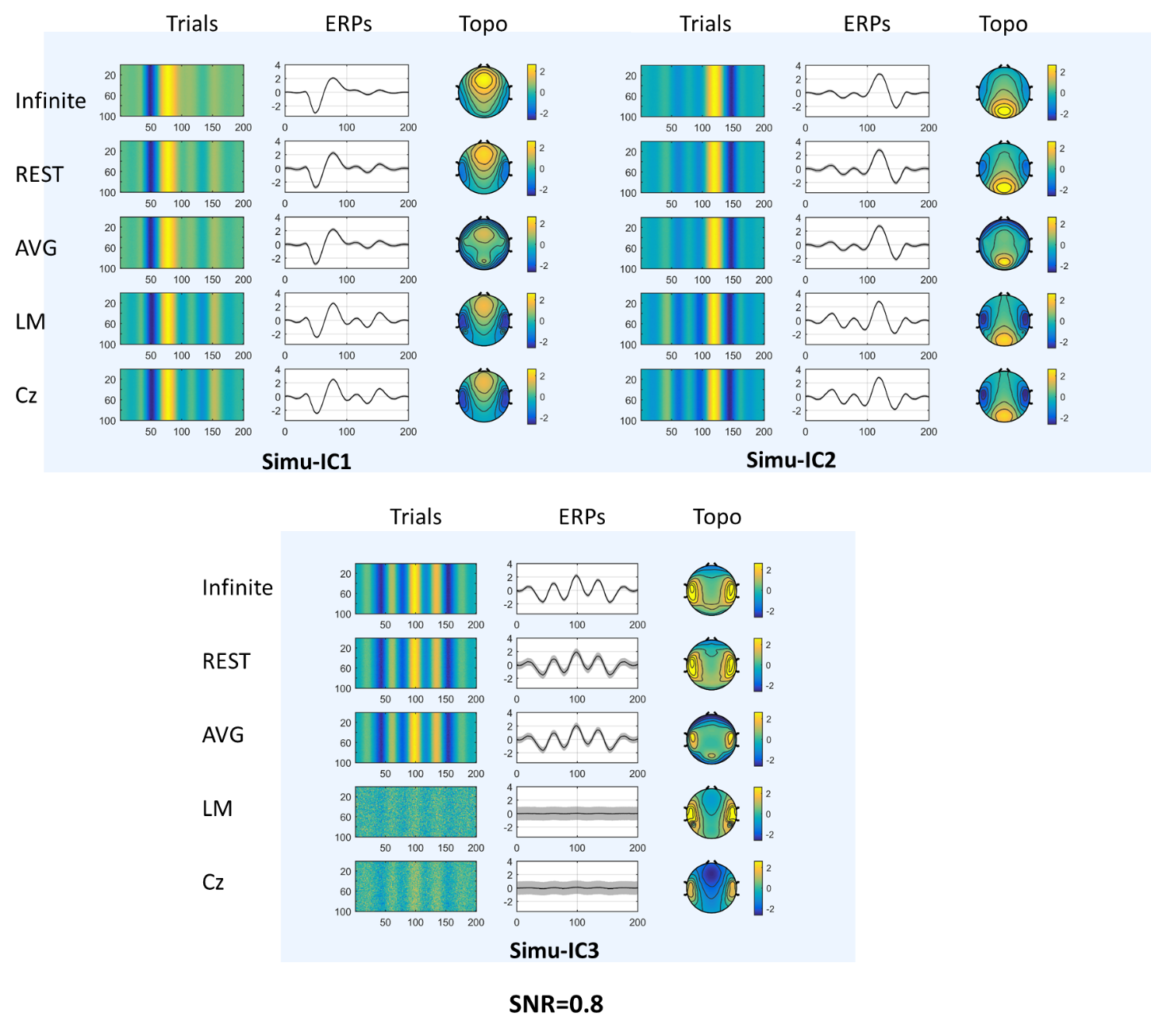


Figure S5: Results of simulation with SNR=0.8


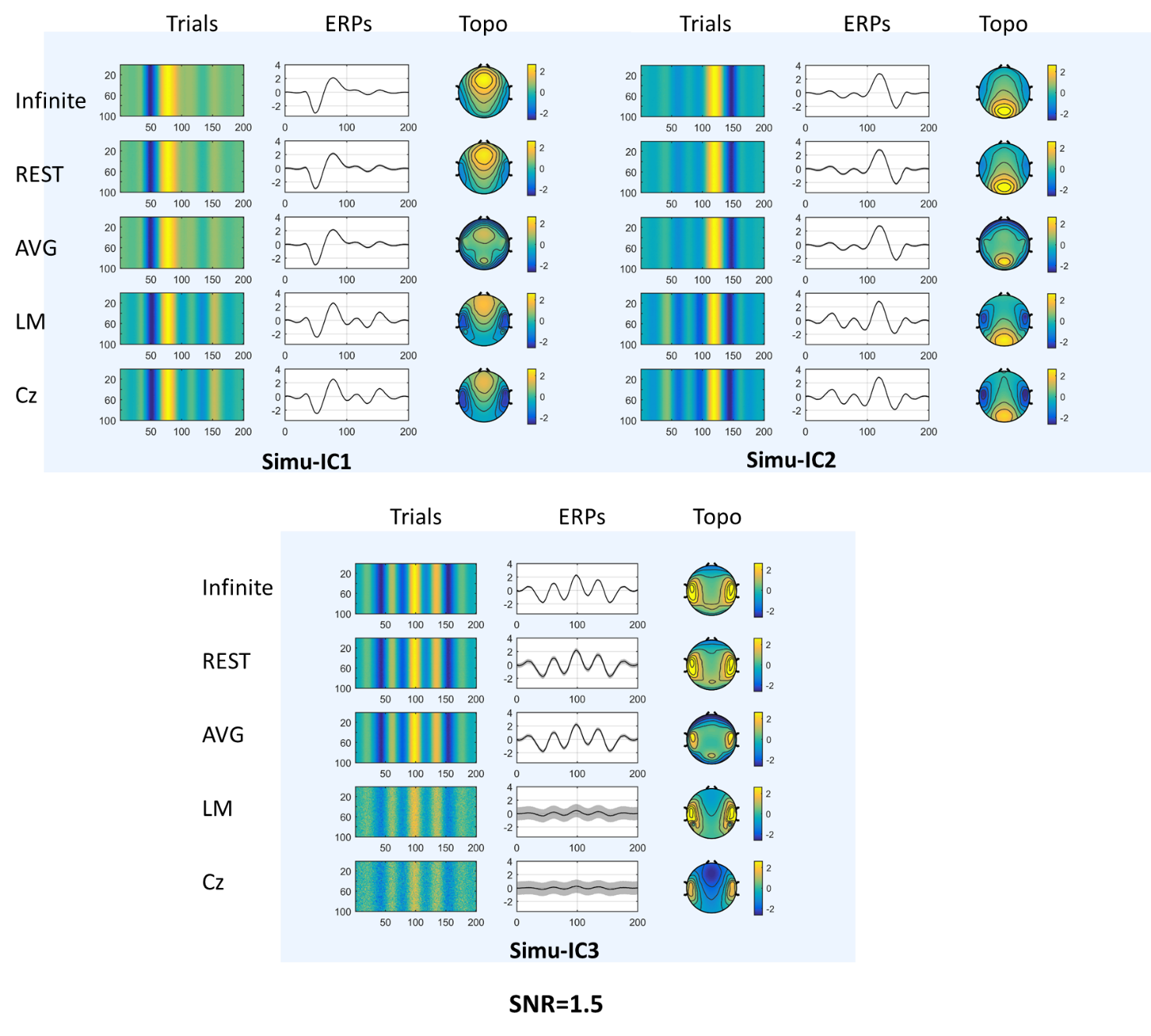


Figure S6: Results of simulation with SNR=1.5


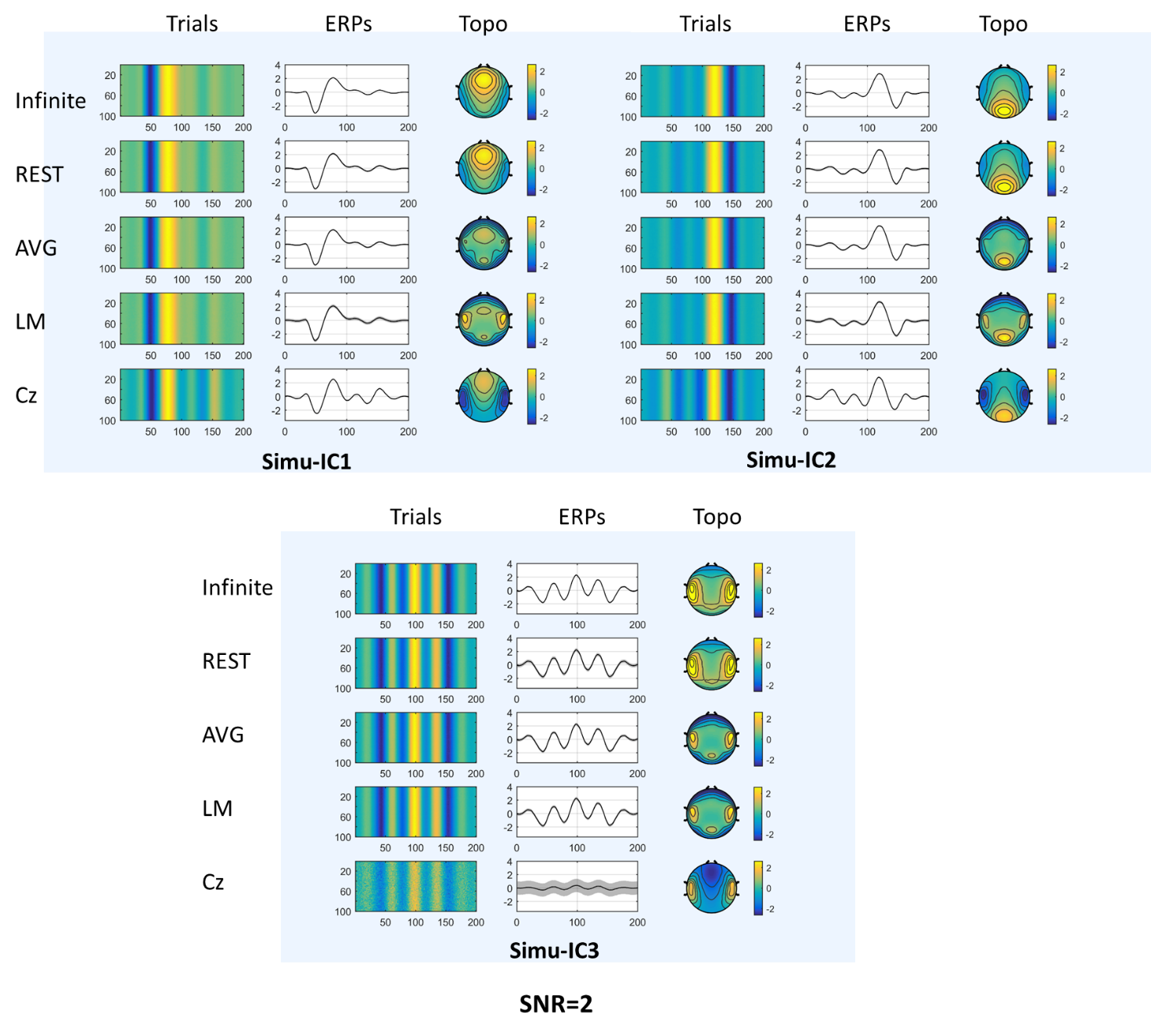


Figure S7: Results of simulation with SNR=2


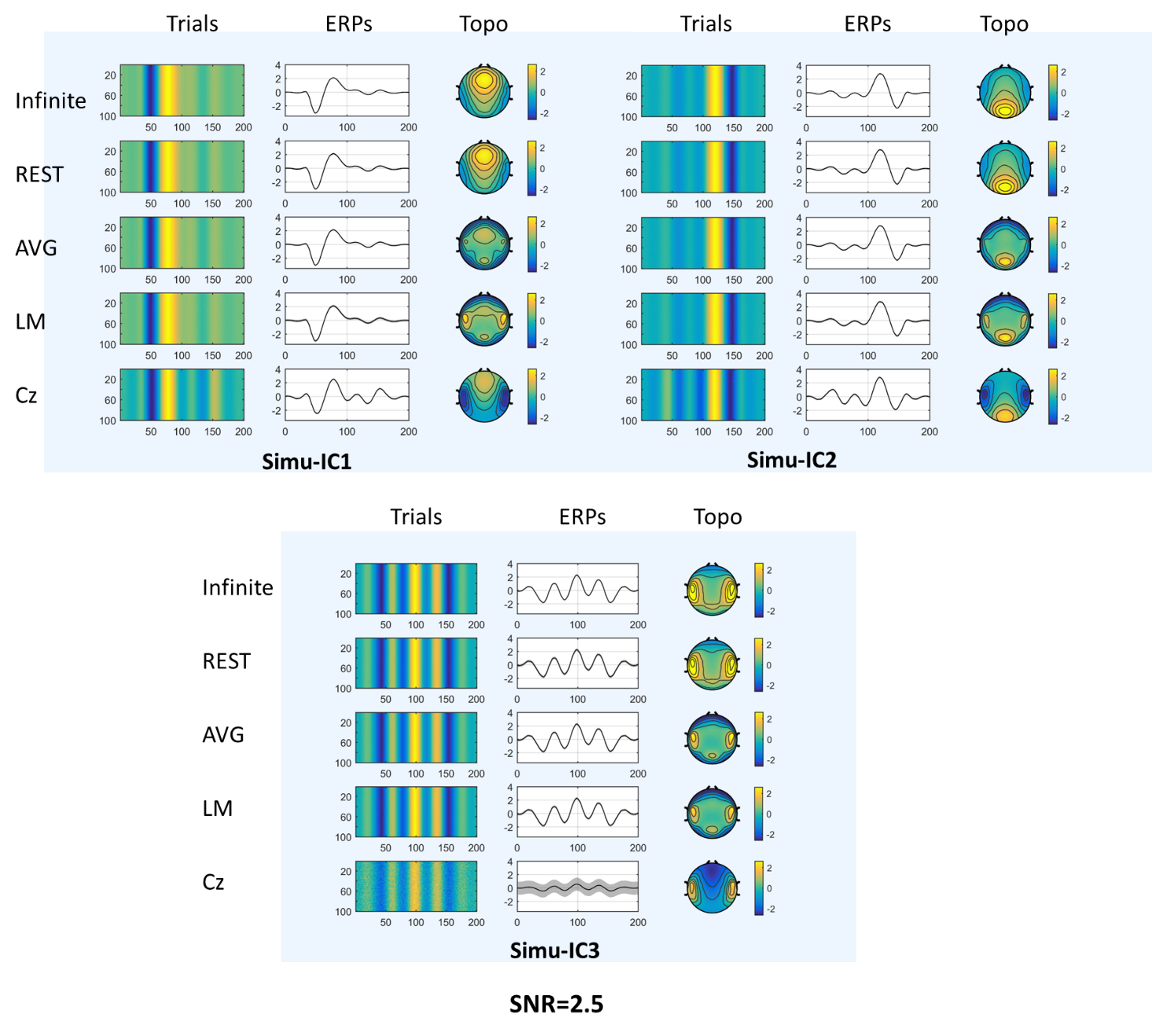


Figure S8: Results of simulation with SNR=2.5


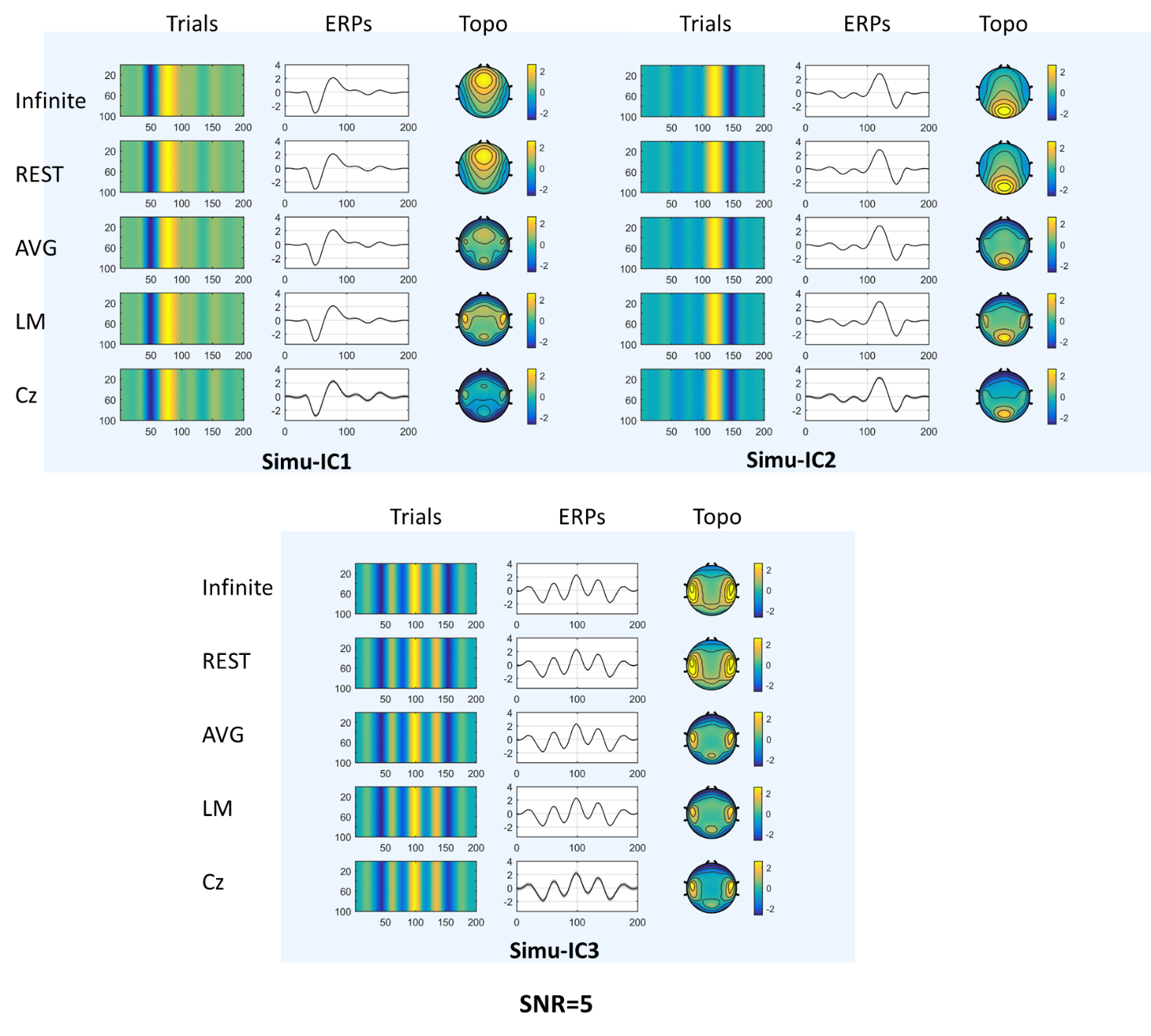


Figure S9: Results of simulation with SNR=5


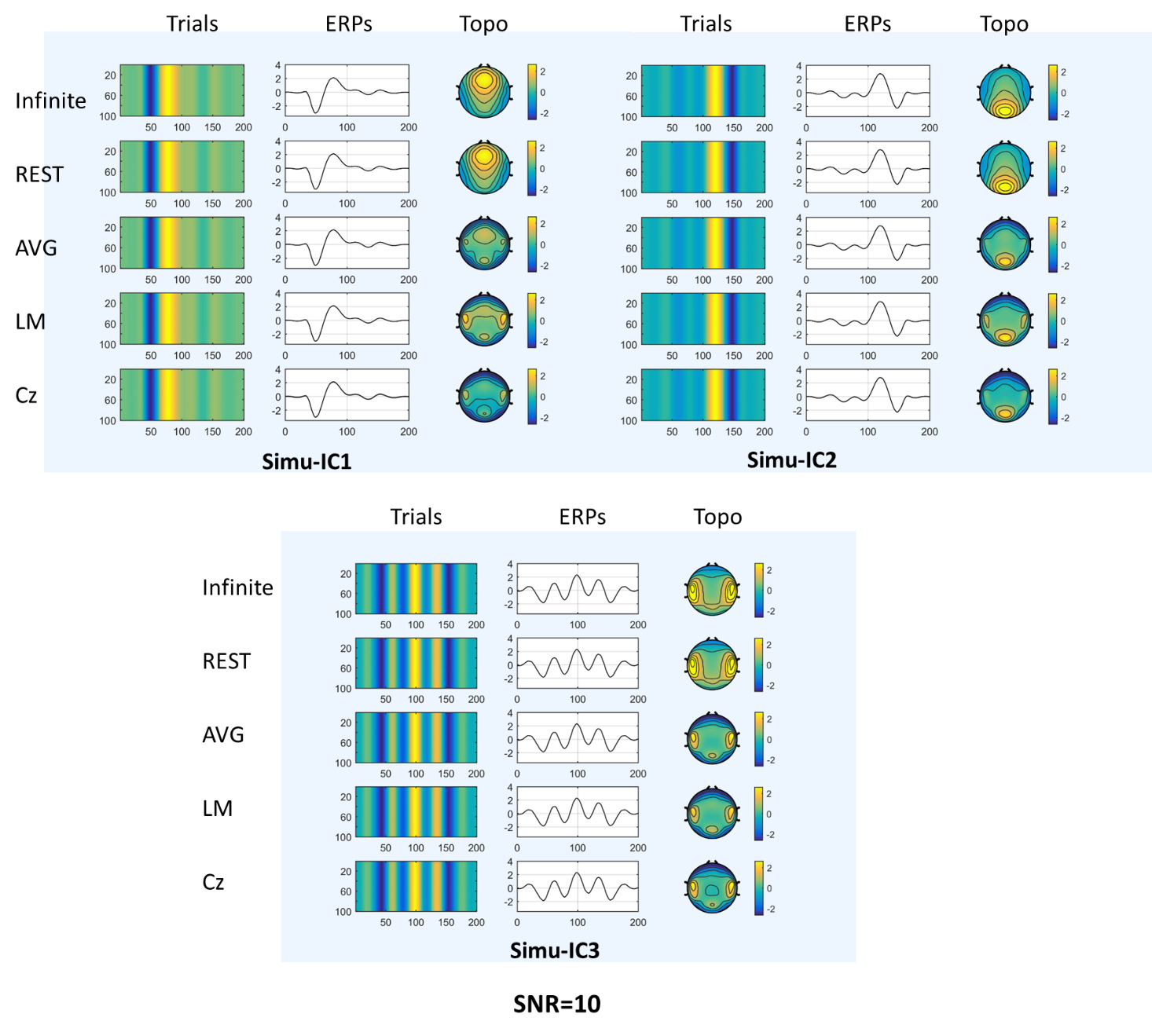


Figure S10: Results of simulation with SNR=10


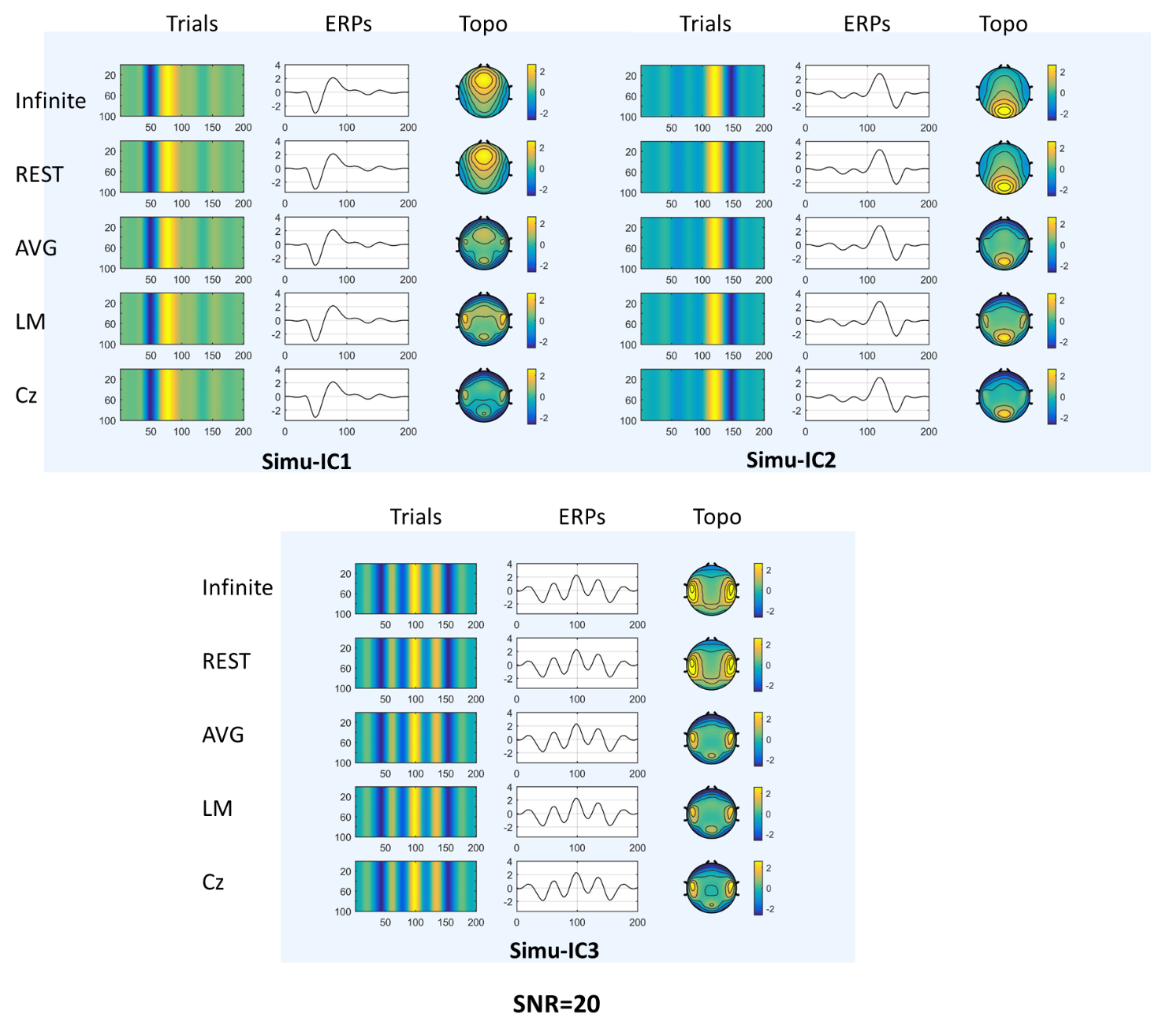


Figure S11: Results of simulation with SNR=20


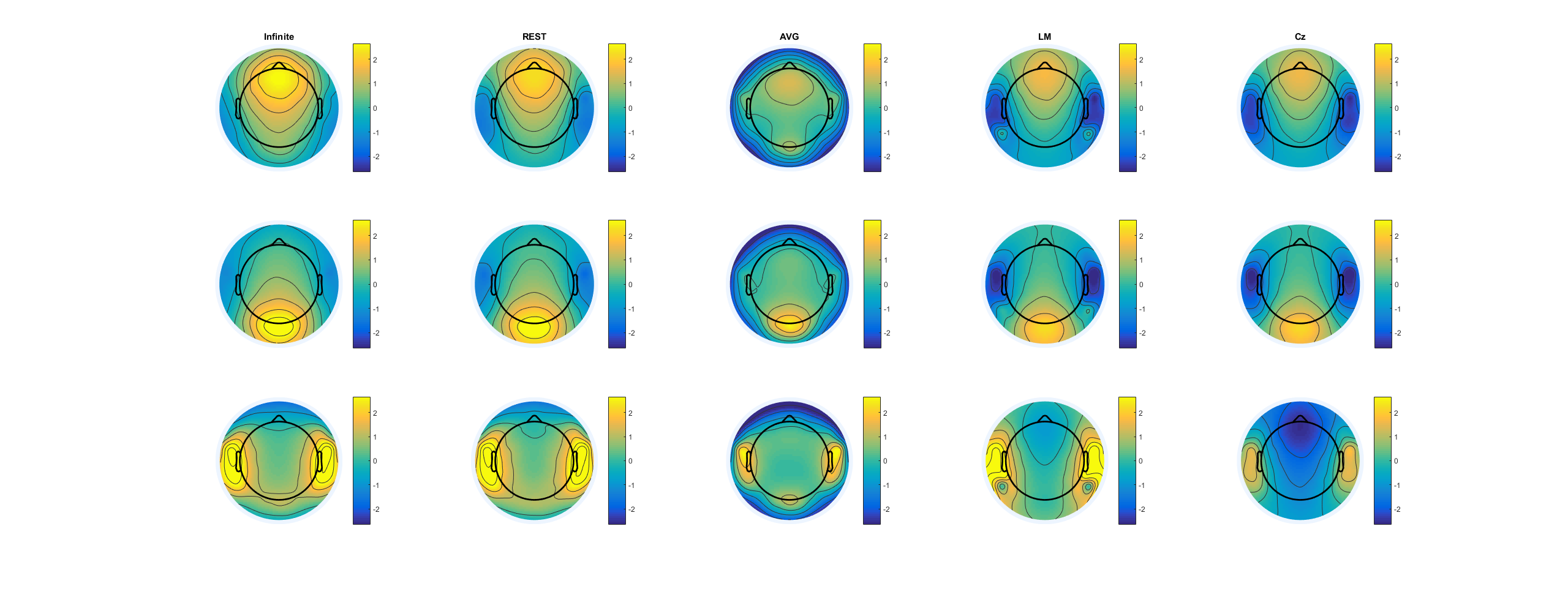


Figure S12: Results of ICA spatial topographies (corresponding to mixing matrix G in ICA model) with SNR=1. Columns from left to right are ICA spatial components of Infinite, REST, AVG, LM and Cz. Rows from upper to low are corresponding to the ICA components (Simulation IC1-IC3).


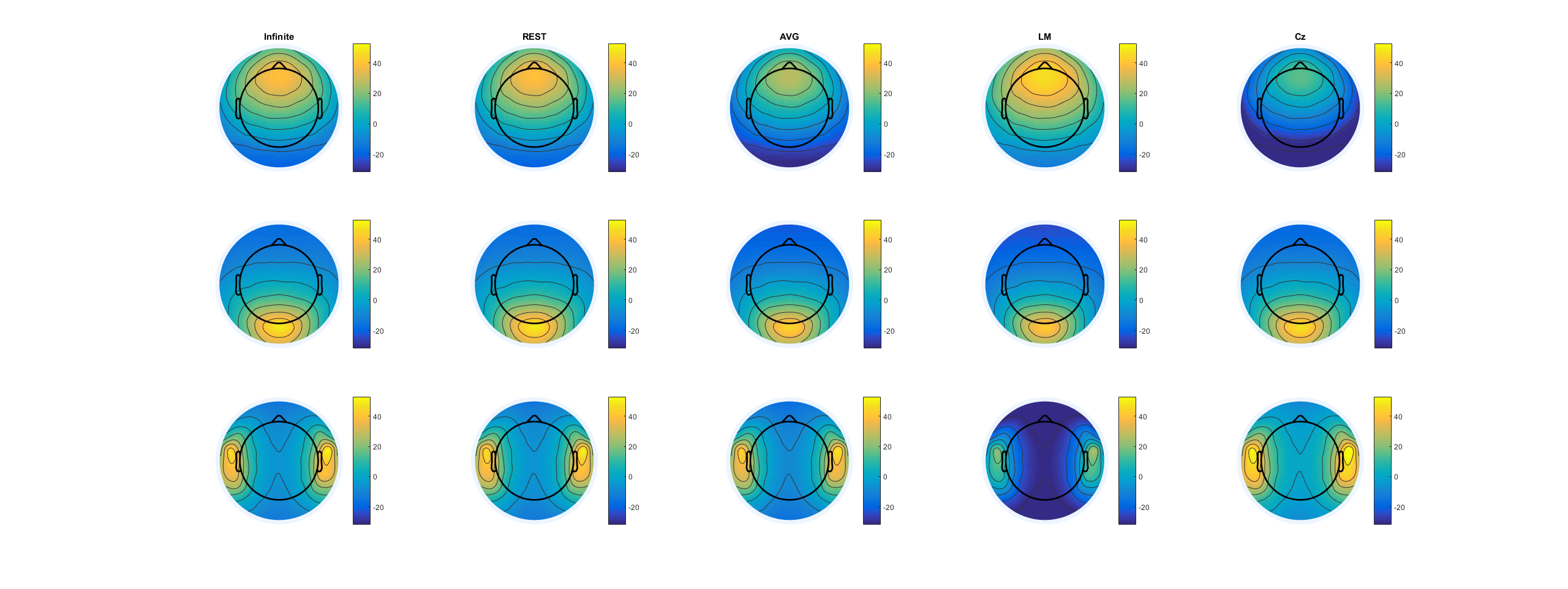


Figure S13: The transform matrix *T^**^=T^*^LS* corresponding to different reference methods respectively. Columns from left to right are corresponding to the Infinite, REST, AVG, LM and Cz. Rows from upper to low are corresponding to the simulation components IC1-IC3.


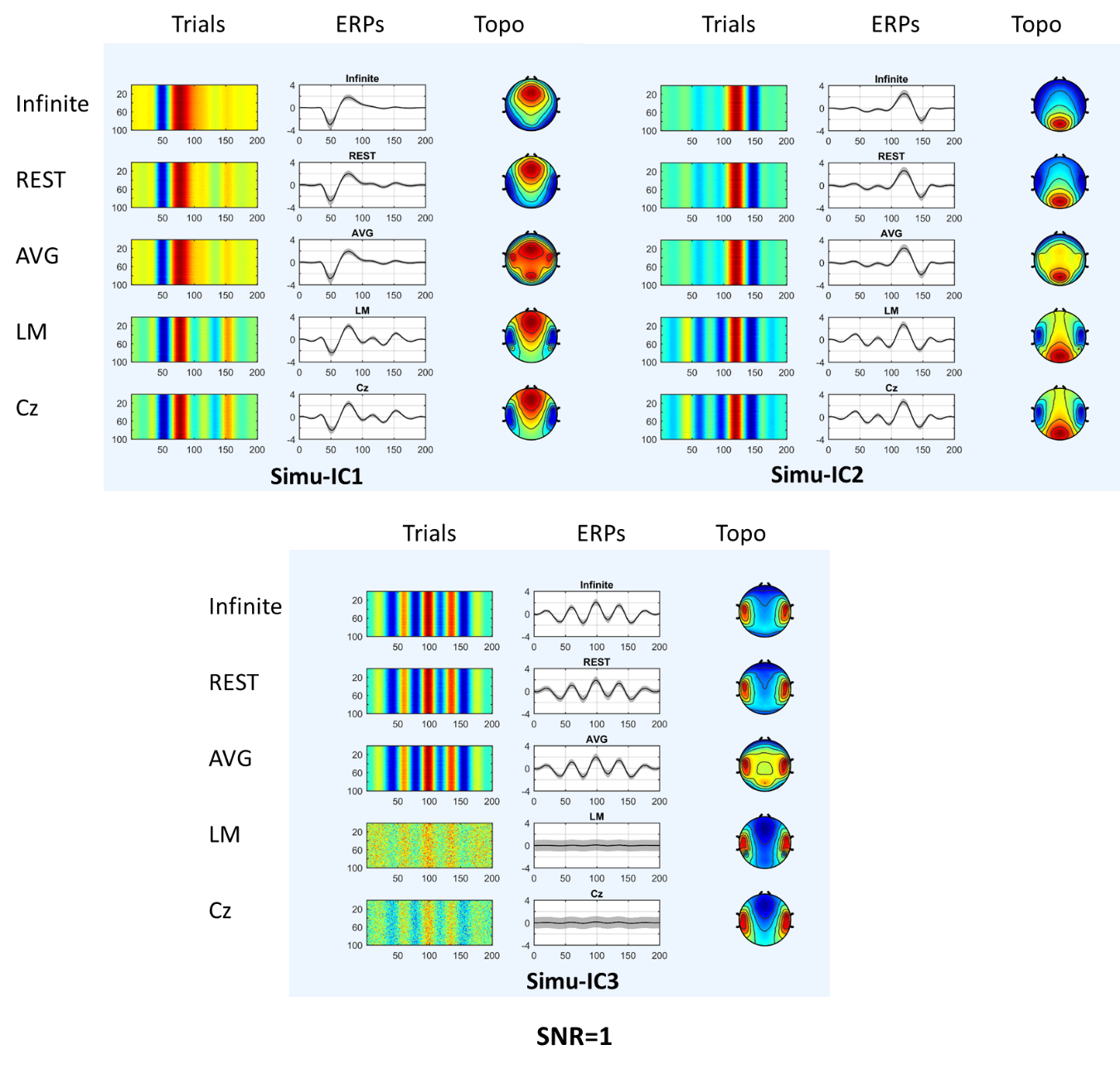


Figure S14: Results of simulation with SNR=1, while the trial amplitudes are independent (uniform distribution from 0.5 to 1.5).


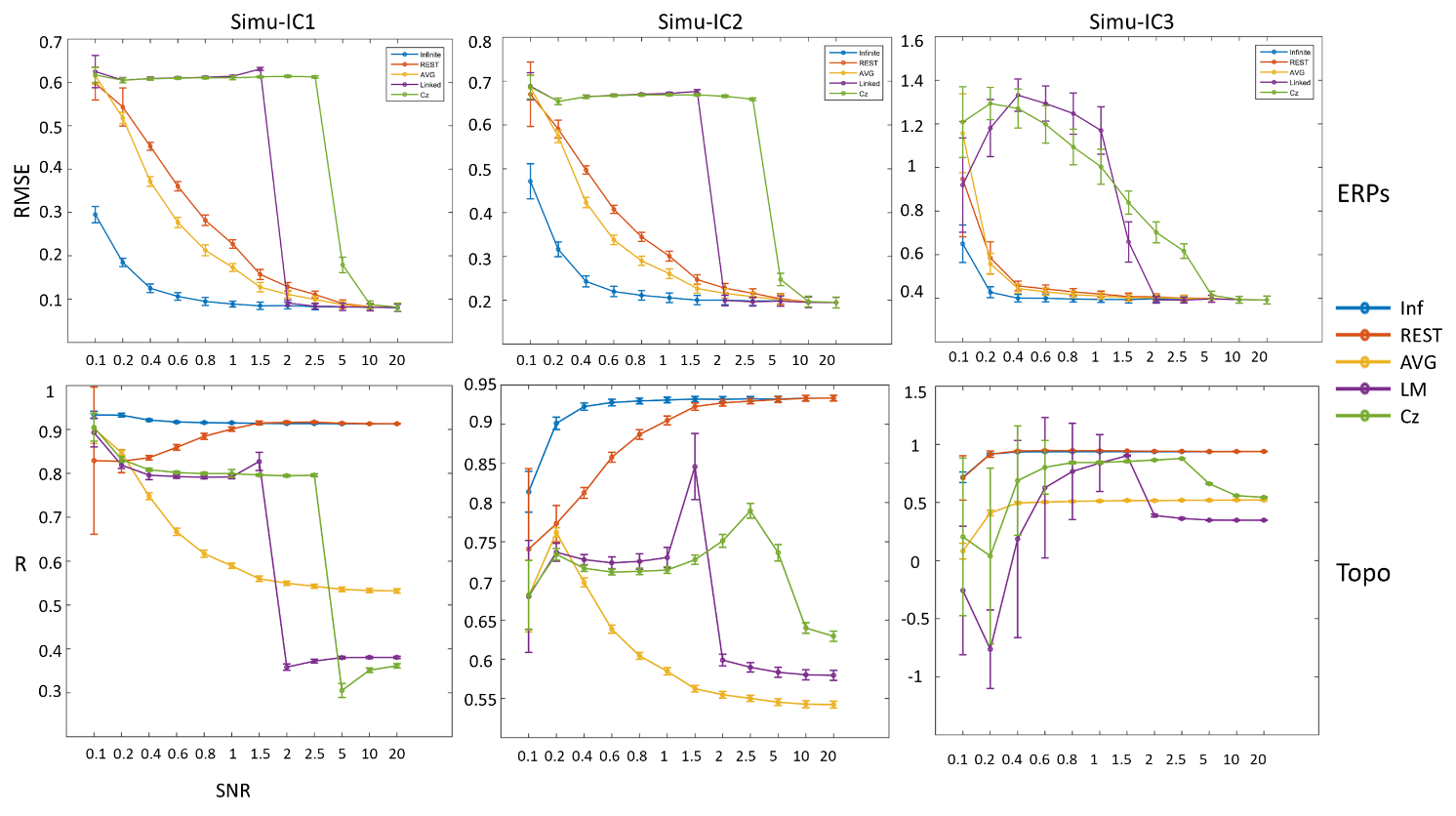


Figure S15: Performances of ICA time courses and topographies with different references (Inf, REST, AVG, LM and Cz) using a range of SNRs from 0.1 to 20, while the trial amplitudes are independent (uniform distribution from 0.5 to 1.5). The vertical axis of the first row is the mean RMSE (with standard deviation) between true noiseless ERPs and ICA-extracted ERPs with a reference, and the vertical axis of the second row is the mean spatial correlation (with standard deviation) between true topographies and ICA-extracted topographies with a reference. Columns from left to right correspond to the ICA components (Simu-IC1, Simu-IC2 and Simu-IC3), and the horizontal axis is the SNR.
